# Cardiovascular protective effect of sodium-glucose cotransporter 2 inhibitors on patients with acute coronary syndrome and type 2 diabetes mellitus: a retrospective study

**DOI:** 10.1186/s12872-023-03542-y

**Published:** 2023-10-07

**Authors:** Jie Chen, Jing Chang, Qiuyue Shi, Xin Li, Ling Wang, Hong Zhao

**Affiliations:** Department of Cardiology, The First Affiliated Hospital of Chongqing Medical University, Chongqing, 400010 China

**Keywords:** Sodium-glucose cotransporter 2 inhibitors, Acute coronary syndrome, Type 2 diabetes mellitus

## Abstract

**Background:**

Acute coronary syndrome (ACS) remains the leading cause of death and disability worldwide, especially when combined with type 2 diabetes mellitus (T2DM). Many multicenter randomized controlled trials have established the cardiovascular benefits of Sodium-Glucose cotransporter 2 inhibitors (SGLT-2i) in patients with T2DM at high cardiovascular risk. However, these studies did not include patients in the early stages of acute coronary events. This study investigated the cardiovascular protective effects of SGLT-2i in patients with ACS and T2DM.

**Methods:**

A total of 232 hospitalized patients with ACS and T2DM were enrolled and divided into two groups based on their hypoglycemic drug treatment: the SGLT-2i and the non-SGLT-2i groups. Kaplan–Meier analysis and Cox regression were used to compare adverse cardiovascular outcomes in both groups.

**Results:**

There were no significant differences in the hospital clinical outcomes between the SGLT-2i and non-SGLT-2i groups. The adverse cardiovascular outcomes did not significantly differ between both groups (hazard ratio (HR) 0.66, 95% confidence interval (CI) 0.35–1.25, *P* = 0.195). Moreover, the rehospitalization rates for ACS or heart failure (HF) were not significantly different between both groups (adjusted HR 0.56, 95%CI 0.28–1.10, *P* = 0.093). When analyzed separately, there was no significant difference in rehospitalizations for ACS (HR 0.87, 95% CI 0.40–1.87, *P* = 0.713). However, the SGLT-2i group showed lower rates of rehospitalizations for HF (adjusted HR 0.20, 95% CI 0.04–0.96, *P* = 0.045). Additionally, there was no significant difference in cardiovascular mortality between both groups (HR 1.75, 95% CI 0.28–10.97, *P* = 0.543). Notably, the SGLT-2i group exhibited a higher angina symptom control rate than the non-SGLT-2i group (adjusted odd ration (OR) 0.45, 95%CI 0.21–0.93,* P* = 0.031).

**Conclusion:**

In recently diagnosed patients with ACS, who have T2DM, early initiation of SGLT-2i was associated with a lower risk of rehospitalization for HF and a higher rate of angina symptom control.

**Supplementary Information:**

The online version contains supplementary material available at 10.1186/s12872-023-03542-y.

## Introduction

Despite advancements in early reperfusion therapy and medical treatments, acute coronary syndrome (ACS) remains a significant contributor to global mortality and disability [1]. Epidemiological data have shown that more than 40% of patients with ACS have diabetes, which has been demonstrated to be an independent predictor of long-term major adverse cardiovascular event in patients with high cardiovascular risk [2]. Additionally, hyperglycemia has been shown to be associated with increased in-hospital mortality in patients undergoing primary percutaneous coronary intervention (PCI) [3], and this association holds true when patients have cardiogenic shock [4, 5]. Patients with ACS and diabetes often present extensive coronary plaque accumulation, larger lipid cores within the plaques, increased macrophage infiltration, and higher plaque calcification levels [6], leading to a higher risk of cardiovascular death [7].

Sodium-glucose cotransporter 2 inhibitors (SGLT-2i) have demonstrated their ability to lower blood glucose levels by reducing glucose reabsorption in the proximal convoluted tubules of the kidney [8]. Many multicenter randomized controlled trials have established the cardiovascular benefits of SGLT-2i in patients with type 2 diabetes mellitus (T2DM) at high cardiovascular risk. However, these studies did not include patients in the early stages of acute coronary events. Given these potential benefits, it is hypothesized that patients with ACS and T2DM may experience advantages from the early initiation of SGLT-2i treatment [9]. To further explore this, we conducted a retrospective study to investigate the effects of early SGLT-2i initiation on in-hospital clinical outcomes and long-term cardiovascular prognosis in patients with ACS and T2DM.

## Methods

### Participants

We retrospectively screened 232 patients admitted to the First Affiliated Hospital of Chongqing Medical University between January 2019 and June 2022. These patients were diagnosed with T2DM and hospitalized for ACS, including unstable angina, non-ST-segment elevated myocardial infarction (NSTEMI), and ST-segment elevated myocardial infarction (STEMI). Unstable angina refers to clinical symptoms suggestive of ACS without biochemical evidence of myocardial infarction. Diagnoses of NSTEMI and STEMI were based on the 4th Universal Definition of myocardial infarction [10]. Patients were divided into two groups according to the hypoglycemic agent used: the SGLT-2i group, if they had been taking SGLT-2i before or during hospitalization, and the non-SGLT-2i group, if they had never used SGLT-2i. Certain exclusion criteria were applied, including age < 18 or > 80 years, a history of refractory heart failure (HF), congenital heart disease, severe valvular heart disease, type 1 diabetes mellitus, cirrhosis with a baseline estimated glomerular filtration rate (eGFR) ≤ 45 mL/min/1.73 m^2^, malignancies, other life-threatening conditions, and datasets with more than 20% missing values. The Ethical Review Board of the First Affiliated Hospital of Chongqing Medical University approved the study protocol.

### Data collection

The patients’ basic information, medical history, auxiliary examinations, and treatment information were collected using the hospital information system. Prognostic data were obtained via telephone calls or case reviews. Endpoints included in-hospital clinical outcomes, adverse cardiovascular outcomes, and angina symptom control rates. In- hospital clinical outcomes included length of hospital stay, occurrence of acute heart failure (AHF), mortality risk of after discharge, new-onset cardiac arrhythmias, and hypotensive events. Adverse cardiovascular outcomes included rehospitalization for ACS or HF and cardiovascular mortality. The control rate of angina symptoms was assessed using the Canadian Cardiovascular Society (CCS) angina classification. Patients who achieved CCS classes I and II were considered to have controlled symptoms. The Global Registry for Acute Coronary Events (GRACE) risk score was used to assess mortality risk after discharge [11]. A GRACE risk score > 118 indicated a high-risk group. New-onset cardiac arrhythmias were defined as a composite of new-onset atrial fibrillation (AF) or ventricular tachycardia/tachyarrhythmia (VT/VF) during hospitalization. Hypotensive events were defined as systolic blood pressure < 90 mmHg or a decrease of > 30 mmHg from the baseline systolic blood pressure.

### Statistical analysis

Statistical analyses were performed using IBM SPSS Statistics for Windows, version 25.0. Continuous variables are presented as means and standard deviations (SD) or medians and interquartile ranges, and categorical variables are presented as numbers and percentages. The chi-square test was used to compare continuous variables between both groups. For categorical variables, the *t*-test or Mann–Whitney U test was used. Kaplan–Meier survival curves, and the log-rank test were used to compare adverse cardiovascular outcomes. A Cox regression model was used to calculate the hazard ratios (HRs) and odds ratios (ORs) were calculated using a logistic regression model. A univariate analysis was performed to identify potential confounders. Variables with *P* < 0.1 were included in the multivariate Cox regression model or multivariate logistic regression model to obtain adjusted HRs or ORs, along with their corresponding *P*-values. Statistical significance was defined as* P* < 0.05.

## Results

### Baseline characteristics

A total of 232 patients were included in this study, with 128 and 104 patients in the SGLT-2i and non-SGLT-2i groups, respectively. Baseline characteristics of the study participants are presented in Table 1. Compared to the non-SGLT-2i group, the SGLT-2i group had a higher prevalence of hypertension (69.5% VS 52.9%, *P* = 0.009). Only 1.7% of the patients had a history of HF before admission. Among all patients, 30.6% had unstable angina, 24.1% had NSTEMI, and 45.3% had STEMI. The Gensini score, which assesses the severity of coronary artery disease (CAD), did not significantly differ between both groups. Regarding the prescription of SGLT-2i, 63.3% of patients received empagliflozin, while 36.7% received dapagliflozin. The median time to SGLT-2i initiation was 3 (1–5) days. There were no significant differences in other medication treatments between the two groups. All patients underwent PCI during hospitalization, with approximately 80% receiving coronary stenting, a similar proportion in both groups. The SGLT-2i group exhibited a slightly larger left atrial diameter (LAD) than the non-SGLT-2i group (33 mm VS 32 mm, *P* = 0.022). However, no significant differences were observed in other echocardiographic parameters or biochemical indicators between both groups.

**Table 1:** Baseline characteristics of study population

	SGLT-2i group (*n* = 128)	Non-SGLT-2i group (*n* = 104)	*P* value
Age, years	64 (56,70)	67 (57,71)	0.325
Male (%)	96 (75.0%)	79 (76.7%)	0.765
BMI, kg/m^2^	24.92 $\pm 3.46$	24.51 $\pm 3.40$	0.384
ACS (%)			0.174
Unstable angina	44 (34.4%)	27 (26.0%)	
NSTEMI	33 (25.8%)	23 (22.1%)	
STEMI	51 (39.8%)	54 (51.9%)	
Smoking (%)	70 (54.7%)	61 (58.7%)	0.545
Old stroke (%)	9 (7.0%)	6 (5.8%)	0.697
Hypertension (%)	89 (69.5%)	55 (52.9%)	0.009
CAD (%)	17 (13.3%)	19 (18.3%)	0.297
Congestive heart failure (%)	4 (3.1%)	0 (0.00%)	0.130
Duration of T2DM, years	1.0 (0,10)	3.5 (0,10)	0.255
High Risk of in-hospital death (%)	39 (30.5%)	39 (37.5%)	0.260
Gensini score	48.0 (24.0,80.0)	47.5 (22.8,82.0)	0.824
Coronary stenting (%)	100 (78.1%)	84 (80.8%)	0.621
**Drugs**			
Antiplatelet therapy (%)	128 (100%)	104 (100%)	
β-Blocker (%)	97 (75.8%)	80 (76.9%)	0.839
Lipid-lowering therapy (%)	128 (100%)	104 (100%)	
ACEi/ARB/ARNi (%)	98 (76.6%)	67 (67.3%)	0.117
CCBs (%)	21 (16.4%)	10 (9.6%)	0.131
Diuretics (%)	25 (19.5%)	24 (23.1%)	0.511
Insulin (%)	31 (24.2%)	27 (26.0%)	0.760
Sulfonylurea (%)	15 (11.7%)	15 (14.4%)	0.542
GLP-1 receptor agonist (%)	15 (11.7%)	9 (8.7%)	0.446
DPP-IV Inhibitors (%)	3 (2.3%)	3 (2.9%)	0.796
Metformin (%)	71 (55.5%)	60 (57.7%)	0.734
Empagliflozin (%)	81 (63.3%)	0 (0%)	
Dapagliflozin (%)	47 (36.7%)	0 (0%)	
**Biochemical indicators**			
HbA1c	7.60 (6.30,8.90)	7.40 (6.63,8.38)	0.635
Scr, mmol/L	74 (62,89)	71 (59,84)	0.169
UA, μmol/L	335 (272,409)	331 (277,390)	0.593
TC, mmol/L	4.36 (3.66,5.04)	4.01 (3.40,4.94)	0.195
HDL, mmol/L	0.96 (0.86,1.12)	0.95 (0.81,1.13)	0.346
LDL, mmol/L	2.54 (1.99,3.19)	2.39 (1.76,3.14)	0.219
LP(α), mmol/L	102 (57,290)	133 (46,391)	0.835
hs-CRP, mg/dL	3.52 (1.68,10.94)	4.53 (1.44,12.45)	0.846
**Echocardiographic**			
LVEF (%)	59 (48,63)	58 (49,63)	0.516
LAD (mm)	33 (31,36)	32 (30,35)	0.022
LVIDd (mm)	48 (45,51)	48 (44,48)	0.969
IVSd (mm)	10 (10,11)	10 (10.11)	0.537
LVPWd (mm)	10 (10,11)	10 (10,11)	0.389

*CAD* Coronary artery disease, *CCB* Calcium Channel Blocker, *ACEi* angiotensin converting enzyme inhibitor, *ARB* angiotensin receptor blocker, *ARNi* Angiotensin receptor-neprilysin inhibitor, *GLP-1* Glucagon Like Peptide 1, *DPP-IV* Dipeptidyl Peptidase IV, *LVEF* left ventricular ejection fraction, *LAD* left atrial diameter, *LVIDd* left ventricular end-diastolic diameter, *IVSd* interventricular septal thickness, *LVPWd* left ventricular posterior wall thickness

### In-hospital clinical outcomes

The in-hospital clinical outcomes of both groups are shown in Table 2. The median length of hospital stay for all patients was 8 days, with no significant difference between both groups. Early initiation of SGLT-2i did not significantly reduce the occurrence of AHF in patients with ACS and T2DM during hospitalization (28.9% VS 26.0%, *P* = 0.618). Approximately 24.6% of patients were classified as high risk, with no significant difference between the two groups (25.8% VS 23.1%, *P* = 0.634). Thirty new-onset cardiac arrhythmias were observed during hospitalization, with no significant differences.

**Table 2:** The In-hospital outcomes

	All (*n* = 232)	SGLT-2i group (*n* = 128)	non-SGLT-2i group (*n* = 104)	*P value*
Hospital stay, days	8 (6–10)	8 (6–10)	8 (6–12)	0.654
High risk of death after discharge	57 (24.6%)	33 (25.8%)	24 (23.1%)	0.634
Acute heart failure	64 (27.7%)	37 (28.9%)	27 (26.0%)	0.618
New-onset cardiac arrhythmias	30 (13.1%)	14 (10.9%)	16 (15.8%)	0.621
Hypotensive events	25 (10.8%)	15 (11.7%)	10 (9.6%)	0.607

### Adverse cardiovascular outcomes

**Fig. 1:**
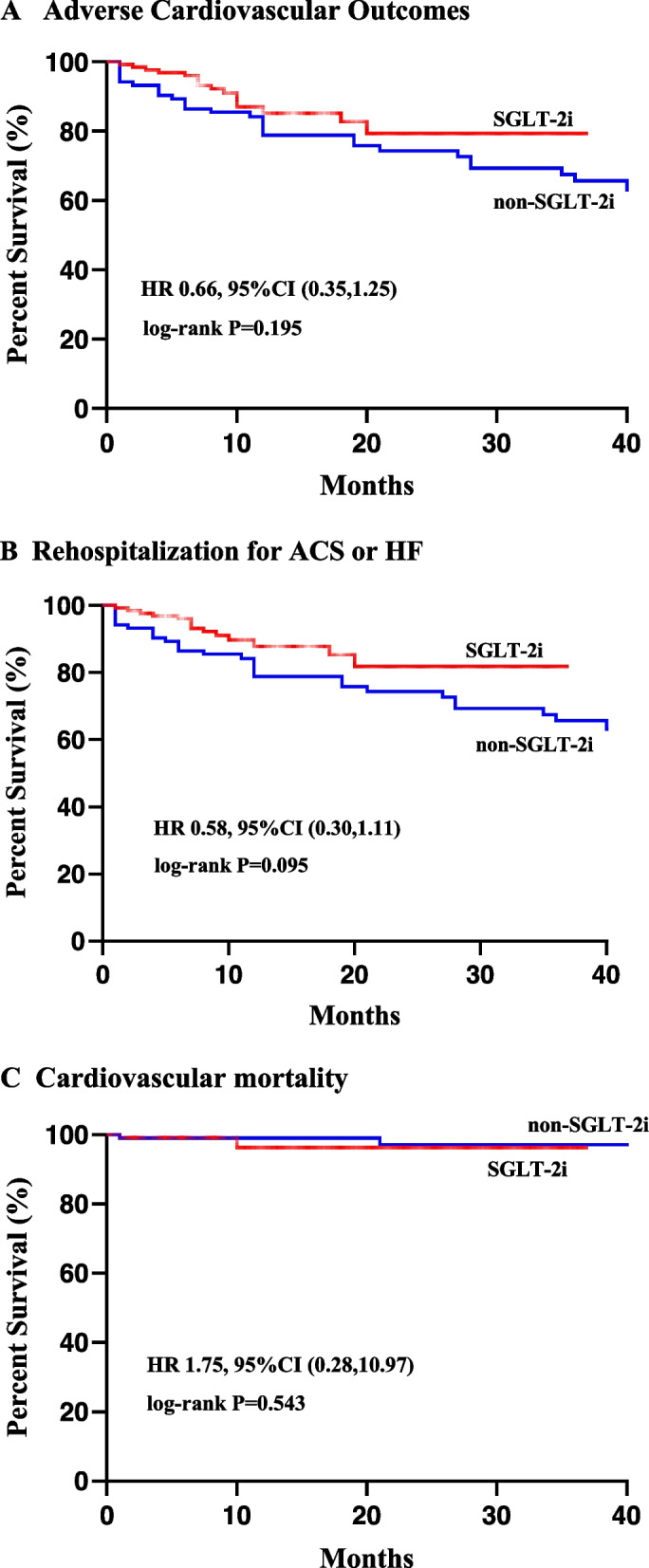
Kaplan-Mayer survival curves of cardiovascular outcomes

To assess the effects of SGLT-2i on adverse cardiovascular outcomes, Kaplan–Meier survival curves were constructed (Fig. 1). The median follow-up period was 10 (8,23) months. During the follow-up period, 46 cardiovascular adverse outcomes were recorded, with 16 (12.5%) in the SGLT-2i group and 30 (28.8%) in the non-SGLT-2i group. However, there was no significant difference in adverse cardiovascular outcomes between both groups (HR 0.66, 95% CI 0.35–1.25,* P* = 0.195).

**Fig. 2:**
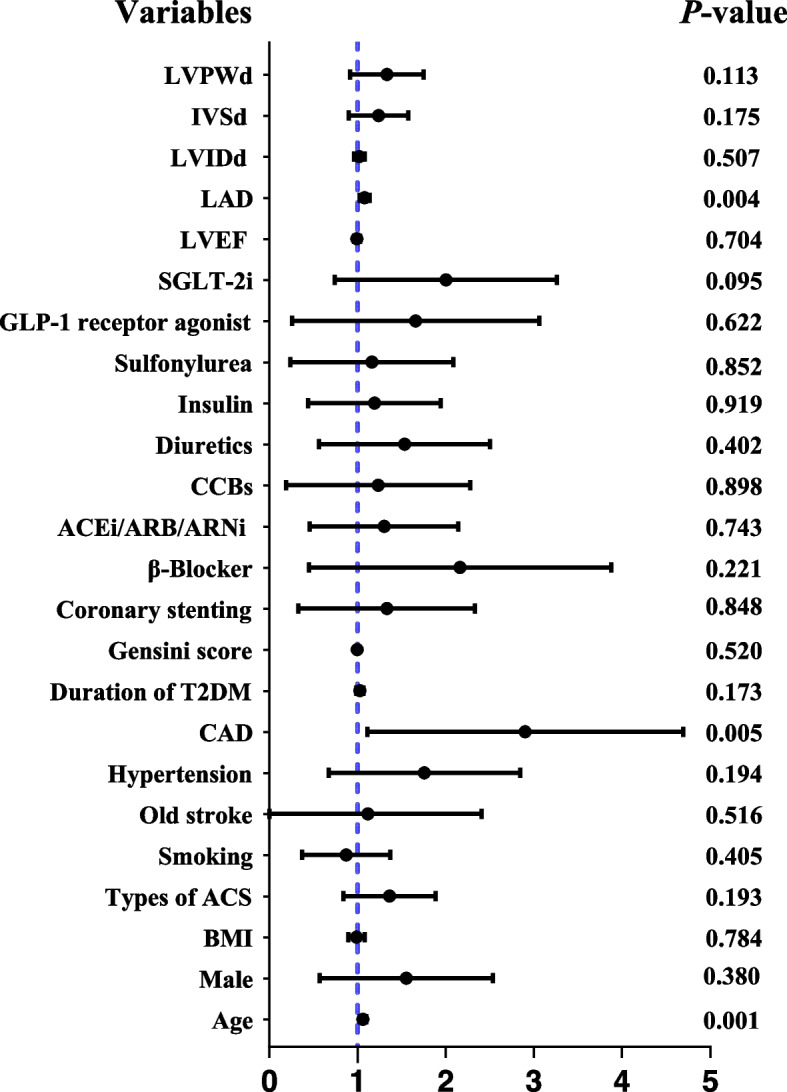
Predictors of rehospitalization. Note: variables which *P* < 0.10 were considered potential confounders, including age, CAD and LAD; Hypertension was considered a potential confounder due to baseline differences

Table 3 presents the evaluation of individual components of adverse cardiovascular outcomes. Univariate analysis found that rehospitalization for ACS or HF was lower in the SGLT-2i group than in the non-SGLT-2i group, though statistical insignificance (HR 0.58, 95%CI 0.30–1.11, *P* < 0.10). After adjusting for potential confounders (Fig. 2), SGLT-2i did not demonstrate a significant reduction in rehospitalization for ACS or HF (adjusted HR 0.56, 95%CI 0.28–1.10, *P* = 0.093). When analyzed separately, SGLT-2i significantly reduces HF rehospitalization after adjusting for potential confounders (adjusted HR 0.20, 95% CI 0.04–0.96, *P* = 0.045). However, there was no significant effect on rehospitalization for ACS. Additionally, there was no significant difference between both groups in terms of cardiovascular mortality (HR 1.75, 95% CI 0.28–10.97, *P* = 0.543). Univariate and multivariate analyses of each component of adverse cardiovascular outcomes are shown in Tables S1-S4 in Additional file 1.

**Table 3:** Each component of adverse cardiovascular outcomes

	**HR (95%CI)**	***P***	HR*(95%CI)	P*
Adverse cardiovascular outcomes	0.66 (0.35,1.25)	0.195		
Rehospitalization for ACS or HF	0.58 (0.30,1.11)	0.095	0.56 (0.28,1.10)	0.093
Rehospitalization for ACS	0.87 (0.40,1.87)	0.713		
Rehospitalization for HF	0.18 (0.04,0.84)	0.014	0.20 (0.04,0.96)	0.045
Cardiovascular mortality	1.75 (0.28,10.97)	0.543		

Multivariate Cox regression models were used to adjust for results with *P* < 0.10; HR* and P*, HR and *P* values after adjusting for potential confounders, including age, CAD, LAD, and hypertension

### Angina symptom control rate

A logistic regression model determined the relationship between SGLT-2i and the angina symptom control rate (Table 4). The multivariate logistic regression model included variables with *P* < 0.10 as potential confounders, including sex, hypertension, CAD, T2DM duration, and LAD (Table S5 in Additional file 1). After adjusting for potential confounders, SGLT-2i was associated with a higher rate of angina symptom control (adjusted OR 0.45, 95%CI 0.21–0.93,* P* = 0.031).

**Table 4:** Angina symptom control rate

	***OR* (95%CI)**	***P***	OR*(95%CI)	P*
Angina Symptom Control Rate	0.51 (0.26,1.03)	0.059	0.45 (0.21,0.93)	0.031

Multivariate logistic regression models; OR* and P*, *OR* and *P* values after adjusting for potential confounders, including sex, hypertension, CAD, Duration of T2DM and LAD

## Discussion

The major findings of this study can be summarized as follows:1. SGLT-2i was not significantly associated with improved in-hospital clinical outcomes. 2. In patients with ACS and T2DM, early initiation of SGLT-2i was not significantly associated with adverse cardiovascular outcomes. Consistency was mainly driven by rehospitalization for ACS and cardiovascular mortality, whereas SGLT-2i reduced rehospitalization for HF. 3. SGLT-2i improved angina symptoms in patients with ACS and T2DM.

SGLT-2i has been shown to significantly reduce myocardial infarct size and improve cardiac function in preclinical animal models of ischemia–reperfusion injury [12]. However, in this study, the early initiation of SGLT-2i was not significantly associated with improved in-hospital clinical outcomes. Clinical studies commonly yield results that differ from those obtained from animal studies. Malignant arrhythmias are a common cause of death during hospitalization for acute myocardial infarction (AMI). The EMBODY trial [13] showed that SGLT-2i can reduce sympathetic activity and enhance parasympathetic activity. Furthermore, an observational study recently reported that early use of SGLT-2i decreased the incidence of AF and VT/VF in patients with AMI during hospitalization [14]. In contrast to the aforementioned studies, this study did not observe a significant effect of SGLT-2i on new-onset cardiac arrhythmias. This discrepancy may be attributed to differences in the study populations. Notably, our study included patients with unstable angina (30.6% of the population) with different pathophysiological mechanisms than those with AMI. Importantly, the early initiation of SGLT-2i, in addition to standard care, did not lead to an increased incidence of hypotensive events in patients with ACS, just as in patients with recent worsening HF [15].

Several cardiovascular outcome trials (CVOTs) [16–19] have demonstrated that SGLT-2i provides additional cardiovascular benefits in patients with T2DM who have multiple risk factors for atherosclerotic cardiovascular disease or established atherosclerotic cardiovascular disease. These benefits are primarily reflected in the lower hospitalization rates for HF. SGLT-2i did not have a significant effect on nonfatal MI. The EMMY trial showed that early initiation of SGLT2i in patients with recent AMI was associated with a significantly greater reduction in the N-terminal pro-hormone of brain natriuretic peptide (NT-proBNP) at 26 weeks after discharge [9], accompanied by a significant improvement in echocardiographic functional and structural parameters. These findings suggest that SGLT-2i may improve long-term cardiovascular prognosis in patients with recent AMI. This viewpoint was further elaborated in a recent observational study [20]. We expanded our study population to include patients with ACS and obtained similar conclusions. The early use of SGLT-2i was associated with a lower hospitalization rate for HF. Furthermore, this study showed that early initiation of SGLT2i improved the angina symptom control rate (CCSI-II) in patients with ACS and T2DM after discharge. The cardio protective effect of SGLT-2i is likely associated with its ability to improve myocardial energy metabolism. SGLT-2i can increase the utilization of glucose and fatty acids by myocardial cells, shifting the myocardial fuel from glucose to ketone bodies [21]. This transformation increases the (adenosine triphosphate) ATP content in myocardial cells and improves cardiac remodeling. Also, SGLT-2i can prevent early myocardial injury in diabetes and mitigate the progression to HF by modulating the JunD/PPAR-γ pathway [22].

Compared to patients who are non-diabetic, patients with diabetes exhibit a significant upregulation of SGLT-2 in the endothelial cells of carotid artery plaques. This was associated with concomitantly higher levels of inflammatory reactions and thinning of the fibrous caps [23]. Drugs that work on the SGLT-2 system, such as SGLT-2i, can exert anti-inflammatory effects, increase the minimum fibrous cap thickness, and reduce lipid deposits, thereby contributing to a more stable atherosclerotic coronary plaque [24]. Despite the positive role of SGLT-2i in increasing the stability of atherosclerotic plaques, we found that the use of SGLT-2i did not further reduce rehospitalization for ACS in addition to antiplatelet and lipid-lowering therapies, which is consistent with the results of CVOTs and CVD-REAL Nordic study of SGLT-2i [25]. Paradoxically, other real-world studies suggest that the use of SGLT-2i is associated with lower rates of MI or stroke [26–28]. A recent retrospective study indicated that SGLT-2i can effectively prevent the incidence of intra-stent restenosis (ISR)-related events in patients with AMI and T2DM [29]. Therefore, further research is necessary to evaluate the effectiveness of SGLT-2i in preventing acute atherosclerotic vascular events. Additionally, we did not find a reduction in cardiovascular mortality with SGLT-2i treatment, potentially because of the small number of cases and the limited observation period used in the study.

Overweight or obesity is associated with coronary risk factors and CAD development. However, recent studies have reported that patients who are overweight and obese have better survival rates after ACS [30, 31], a phenomenon known as the "obesity paradox." Although we did not observe an independent association between lower body mass index (BMI) and higher cardiovascular mortality, we found that patients who experienced cardiovascular death events had lower BMI indices (Table S4 in the Additional file 1). Age, cancer, chronic inflammatory disease, and diastolic and systolic HF may be the underlying reasons for the higher mortality risk in patients who are low-weight. Additionally, leptin and adiponectin produced by adipose tissue may protect against atherosclerosis and improve the ischemic state of the myocardium.

Patients with ACS are at high risk of arrhythmias, cardiovascular death, recurrent MI, and chronic heart failure, especially when combined with T2DM. Therefore, there is still motivation to discover new therapeutic approaches to improve in-hospital and long-term outcomes of patients with ACS. SGLT-2i has emerged as an established treatment for patients with HF or T2DM at high cardiovascular risk [32]. However, patients with recently diagnosed ACS have been relatively understudied in SGLT-2i cardiovascular outcome trials. Considering the complex pathophysiological changes and impact of treatment measures, it is unclear whether the results (from an efficacy and safety perspective) are consistent when initiated early in patients with recently diagnosed ACS.

In addition to developing new treatment measures, digital health interventions (DHIs) have shown promising prospects in the management of CAD [33], especially during the coronavirus disease pandemic when remote healthcare services have been widely used. DHIs can provide closer follow-up and effective secondary prevention in patients with ACS through various means, such as mobile text messages, voice messages, video clips, telephone calls, video conferences, mobile applications, and smartwatches. Over the past decade, artificial intelligence (AI) systems have demonstrated their effectiveness in CAD management [34]. AI can examine vast volumes of patient data to predict the likelihood of CAD, assist doctors in determining the most suitable treatment options, and estimate the results of interventional treatment for patients with CAD. Digitization of the health industry contributes to the provision of improved medical services, especially for the management of chronic diseases such as CAD.

### Limitations

This study has a few limitations. First, since this was a retrospective study, only preliminary results could be obtained, and further confirmation of these findings would require large-sample prospective randomized controlled trials. Second, insufficient data were available to analyze the effect of SGLT-2i on in-hospital mortality, and the analysis of long-term cardiovascular mortality was also insufficient owing to the small number of patients enrolled. Third, due to the small number of cases, we did not perform important subgroup analyses, such as those based on the presence or absence of HF or different types of ACS. Finally, insufficient clinical data prevented the evaluation of adverse effects other than hypotension. This study did not assess adverse effects such as acute renal injury and ketoacidosis, which may occur in the early stages following ACS.

### Future directions

The cardiovascular protective effects of SGLT-2i may extend to patients with ACS and T2DM. Combined with recent clinical trial data, patients with ACS but not T2DM may also benefit from the early initiation of SGLT-2i. The cardiovascular protective effects of SGLT-2i in ACS, with or without T2DM, should be further evaluated in future multicenter clinical trials. Additionally, it is important to assess the safety of early use of SGLT-2i in patients with ACS, considering factors such as other medical therapies, hemodynamic status, early reperfusion therapy, and the use of contrast agents, which can affect the safety of early initiation of SGLT-2i in patients with ACS.

## Conclusion

The early initiation of SGLT-2i can reduce rehospitalization for HF and improve angina symptom in patients with ACS and T2DM. These findings of present study suggest that the cardiovascular protective effects of SGLT-2i may extend to patients with ACS and T2DM. However, further clinical trials are imperative to validate these findings.

### Supplementary Information

**Additional file 1:** **Table S1. **Univariate and multivariate analyses of adverse cardiovascular outcomes. **Table S2. **Univariate and multivariate analyses of rehospitalization for ACS or HF. **Table S3. **Multivariate analyses of each component of rehospitalization. **Table S4. **Univariate and multivariate analyses of cardiovascular mortality. **Table S5. **Univariate and multivariate analyses of angina symptom control rate.

## Data Availability

All participants were enrolled in the Department of Cardiology, the First Affiliated Hospital of Chongqing Medical University. De-identified Data from this article will be shared upon reasonable request from the corresponding author.

## Abbreviations

ACS: Acute coronary syndrome
AF: Atrial fibrillation
AHF: Acute heart failure
AI: Artificial intelligence
AMI: Acute myocardial infarction
BMI: Body mass index
CAD: Coronary artery disease
CCS: Canadian Cardiovascular Society
CI: Confidence interval
DHI: Digital health intervention
eGFR: Estimated glomerular filtration rate
GRACE: Global Registry for Acute Coronary Events
HF: Heart failure
HR: Hazard ratios
LAD: Left atrial diameter
MI: Myocardial infarction
NSTEMI: Non-ST-segment elevated myocardial infarction
OR: Odds ratios
PCI: Percutaneous coronary intervention
SGLT-2: Sodium-glucose cotransporter 2
SGLT-2i: Sodium-glucose cotransporter 2 inhibitors
STEMI: ST-segment elevated myocardial infarction
T2DM: Type 2 diabetes mellitus
VT/VF: Ventricular tachycardia/ tachyarrhythmia

## References

[1] GBD 2019 Diseases and Injuries Collaborators. Global burden of 369 diseases and injuries in 204 countries and territories, 1990-2019: a systematic analysis for the Global Burden of Disease Study 2019. Lancet. 2020;396(10258):1204–22. 10.1016/S0140-6736(20)30925-9 PMC7567026 33069326

[2] Hayıroğlu Mİ, Çınar T, Çiçek V, Palice A, Ayhan G, Tekkeşin Aİ (2022). The triglyceride-glucose index can predict long-term major adverse cardiovascular events in turkish patients with high cardiovascular risk. J Lipid Atheroscler.

[3] Ekmekci A, Cicek G, Uluganyan M, Gungor B, Osman F, Ozcan KS, Bozbay M, Ertas G, Zencirci A, Sayar N (2014). Admission hyperglycemia predicts inhospital mortality and major adverse cardiac events after primary percutaneous coronary intervention in patients without diabetes mellitus. Angiology.

[4] Hayıroğlu Mİ, Çanga Y, Yıldırımtürk Ö, Bozbeyoğlu E, Gümüşdağ A, Uzun AO, Kalenderoğlu K, Keskin M, Çinier G, Acarel M (2018). Clinical characteristics and outcomes of acute coronary syndrome patients with intra-aortic balloon pump inserted in intensive cardiac care unit of a tertiary clinic. Turk Kardiyol Dern Ars.

[5] Hayıroğlu Mİ, Keskin M, Uzun AO, Yıldırım Dİ, Kaya A, Çinier G, Bozbeyoğlu E, Yıldırımtürk Ö, Kozan Ö, Pehlivanoğlu S (2019). Predictors of in-hospital mortality in patients with st-segment elevation myocardial infarction complicated with cardiogenic shock. Heart Lung Circ.

[6] Yahagi K, Kolodgie FD, Lutter C, Mori H, Romero ME, Finn AV, Virmani R (2017). Pathology of human coronary and carotid artery atherosclerosis and vascular calcification in diabetes mellitus. Arterioscler Thromb Vasc Biol.

[7] Ding Q, Funk M, Spatz ES, Lin H, Batten J, Wu E, Whittemore R (2022). Sex-specific impact of diabetes on all-cause mortality among adults with acute myocardial infarction: an updated systematic review and meta-analysis, 1988–2021. Front Endocrinol (Lausanne).

[8] Scheen AJ (2015). Pharmacodynamics, efficacy and safety of sodium-glucose co-transporter type 2 (SGLT2) inhibitors for the treatment of type 2 diabetes mellitus. Drugs.

[9] von Lewinski D, Kolesnik E, Tripolt NJ, Pferschy PN, Benedikt M, Wallner M, Alber H, Berger R, Lichtenauer M, Saely CH (2022). Empagliflozin in acute myocardial infarction: the EMMY trial. Eur Heart J.

[10] Thygesen K, Alpert JS, Jaffe AS, Chaitman BR, Bax JJ, Morrow DA, White HD (2018). Fourth universal definition of myocardial infarction (2018). Circulation.

[11] Fox KAA, Dabbous OH, Goldberg RJ, Pieper KS, Eagle KA, Van de Werf F, Avezum A, Goodman SG, Flather MD, Anderson FA (2006). Prediction of risk of death and myocardial infarction in the six months after presentation with acute coronary syndrome: prospective multinational observational study (GRACE). BMJ.

[12] Sayour AA, Celeng C, Oláh A, Ruppert M, Merkely B, Radovits T (2021). Sodium-glucose cotransporter 2 inhibitors reduce myocardial infarct size in preclinical animal models of myocardial ischaemia-reperfusion injury: a meta-analysis. Diabetologia.

[13] Shimizu W, Kubota Y, Hoshika Y, Mozawa K, Tara S, Tokita Y, Yodogawa K, Iwasaki Y-K, Yamamoto T, Takano H (2020). Effects of empagliflozin versus placebo on cardiac sympathetic activity in acute myocardial infarction patients with type 2 diabetes mellitus: the EMBODY trial. Cardiovasc Diabetol.

[14] Cesaro A, Gragnano F, Paolisso P, Bergamaschi L, Gallinoro E, Sardu C, Mileva N, Foà A, Armillotta M, Sansonetti A (2022). In-hospital arrhythmic burden reduction in diabetic patients with acute myocardial infarction treated with SGLT2-inhibitors: insights from the SGLT2-I AMI PROTECT study. Front Cardiovasc Med.

[15] Bhatt DL, Szarek M, Steg PG, Cannon CP, Leiter LA, McGuire DK, Lewis JB, Riddle MC, Voors AA, Metra M (2021). Sotagliflozin in patients with diabetes and recent worsening heart failure. N Engl J Med.

[16] Sarafidis PA, Tsapas A (2016). Empagliflozin, cardiovascular outcomes, and mortality in type 2 diabetes. N Engl J Med.

[17] Neal B, Perkovic V, Mahaffey KW, de Zeeuw D, Fulcher G, Erondu N, Shaw W, Law G, Desai M, Matthews DR (2017). Canagliflozin and cardiovascular and renal events in type 2 diabetes. N Engl J Med.

[18] Wiviott SD, Raz I, Bonaca MP, Mosenzon O, Kato ET, Cahn A, Silverman MG, Zelniker TA, Kuder JF, Murphy SA (2019). Dapagliflozin and cardiovascular outcomes in type 2 diabetes. N Engl J Med.

[19] Cannon CP, Pratley R, Dagogo-Jack S, Mancuso J, Huyck S, Masiukiewicz U, Charbonnel B, Frederich R, Gallo S, Cosentino F (2020). Cardiovascular outcomes with ertugliflozin in type 2 diabetes. N Engl J Med.

[20] Kwon O, Myong J-P, Lee Y, Choi Y-J, Yi JE, Seo SM, Jang S-W, Kim PJ, Lee J-M (2023). Sodium-glucose cotransporter-2 inhibitors after acute myocardial infarction in patients with type 2 diabetes: a population-based investigation. J Am Heart Assoc.

[21] Santos-Gallego CG, Requena-Ibanez JA, San Antonio R, Ishikawa K, Watanabe S, Picatoste B, Flores E, Garcia-Ropero A, Sanz J, Hajjar RJ (2019). Empagliflozin ameliorates adverse left ventricular remodeling in nondiabetic heart failure by enhancing myocardial energetics. J Am Coll Cardiol.

[22] Marfella R, D'Onofrio N, Trotta MC, Sardu C, Scisciola L, Amarelli C, Balestrieri ML, Grimaldi V, Mansueto G, Esposito S (2022). Sodium/glucose cotransporter 2 (SGLT2) inhibitors improve cardiac function by reducing JunD expression in human diabetic hearts. Metabolism.

[23] D'Onofrio N, Sardu C, Trotta MC, Scisciola L, Turriziani F, Ferraraccio F, Panarese I, Petrella L, Fanelli M, Modugno P (2021). Sodium-glucose co-transporter2 expression and inflammatory activity in diabetic atherosclerotic plaques: effects of sodium-glucose co-transporter2 inhibitor treatment. Mol Metab.

[24] Sardu C, Trotta MC, Sasso FC, Sacra C, Carpinella G, Mauro C, Minicucci F, Calabrò P, D’Amico M, D’Ascenzo F (2023). SGLT2-inhibitors effects on the coronary fibrous cap thickness and MACEs in diabetic patients with inducible myocardial ischemia and multi vessels non-obstructive coronary artery stenosis. Cardiovasc Diabetol.

[25] Birkeland KI, Jørgensen ME, Carstensen B, Persson F, Gulseth HL, Thuresson M, Fenici P, Nathanson D, Nyström T, Eriksson JW (2017). Cardiovascular mortality and morbidity in patients with type 2 diabetes following initiation of sodium-glucose co-transporter-2 inhibitors versus other glucose-lowering drugs (CVD-REAL Nordic): a multinational observational analysis. Lancet Diabetes Endocrinol.

[26] Khunti K, Kosiborod M, Kim DJ, Kohsaka S, Lam CSP, Goh S-Y, Chiang C-E, Shaw JE, Cavender MA, Tangri N (2021). Cardiovascular outcomes with sodium-glucose cotransporter-2 inhibitors vs other glucose-lowering drugs in 13 countries across three continents: analysis of CVD-REAL data. Cardiovasc Diabetol.

[27] Kosiborod M, Birkeland KI, Cavender MA, Fu AZ, Wilding JP, Khunti K, Holl RW, Norhammar A, Jørgensen ME, Wittbrodt ET (2018). Rates of myocardial infarction and stroke in patients initiating treatment with SGLT2-inhibitors versus other glucose-lowering agents in real-world clinical practice: Results from the CVD-REAL study. Diabetes Obes Metab.

[28] Kosiborod M, Lam CSP, Kohsaka S, Kim DJ, Karasik A, Shaw J, Tangri N, Goh S-Y, Thuresson M, Chen H (2018). Cardiovascular events associated with SGLT-2 inhibitors versus other glucose-lowering drugs: the CVD-REAL 2 study. J Am Coll Cardiol.

[29] Marfella R, Sardu C, D'Onofrio N, Fumagalli C, Scisciola L, Sasso FC, Siniscalchi M, Marfella LV, D'Andrea D, Minicucci F (2023). SGLT-2 inhibitors and in-stent restenosis-related events after acute myocardial infarction: an observational study in patients with type 2 diabetes. BMC Med.

[30] Şaylık F, Çınar T, Hayıroğlu Mİ (2023). Effect of the obesity paradox on mortality in patients with acute coronary syndrome: a comprehensive meta-analysis of the literature. Balkan Med J.

[31] Fukuoka S, Kurita T, Dohi K, Masuda J, Seko T, Tanigawa T, Saito Y, Kakimoto H, Makino K, Ito M (2019). Untangling the obesity paradox in patients with acute myocardial infarction after primary percutaneous coronary intervention (detail analysis by age). Int J Cardiol.

[32] Heidenreich PA, Bozkurt B, Aguilar D, Allen LA, Byun JJ, Colvin MM, Deswal A, Drazner MH, Dunlay SM, Evers LR (2022). 2022 AHA/ACC/HFSA guideline for the management of heart failure: executive summary: a report of the american college of cardiology/american heart association joint committee on clinical practice guidelines. Circulation.

[33] Şaylık F, Çınar T, Hayıroğlu Mİ, Tekkeşin Aİ (2023). Digital health interventions in patient management following acute coronary syndrome: a meta-analysis of the literature. Anatol J Cardiol.

[34] Hayıroğlu Mİ, Altay S (2023). The role of artificial intelligence in coronary artery disease and atrial fibrillation. Balkan Med J.

