# Supplementary material for: Cardiovascular protective effect of sodium-glucose cotransporter 2 inhibitors on patients with acute coronary syndrome and type 2 diabetes mellitus: a retrospective study
Source: BMC Cardiovasc Disord. 2023 Oct 7;23:495. doi: 10.1186/s12872-023-03542-y (PMC10559512; doi:10.1186/s12872-023-03542-y)
Supplement: Supplementary file 1 — Additional file 1: Table S1. Univariate and multivariate analyses of adverse cardiovascular outcomes. Table S2. Univariate and multivariate analyses of rehospitalization for ACS or HF. Table S3. Multivariate analyses of each component of rehospitalization. Table S4. Univariate and multivariate analyses of cardiovascular mortality. Table S5. Univariate and multivariate analyses of angina symptom control rate. [file 12872_2023_3542_MOESM1_ESM.docx]

**Table S1 Univariate and multivariate analyses of adverse cardiovascular outcomes**

|  | Univariate Analysis | | Multivariate Analysis | | |
| --- | --- | --- | --- | --- | --- |
|  | *P*-value | 95% CI | *P*-value | 95% CI | |
| Age, years | 0.001 | 1.022-1.097 | 0.008 | 1.012-1.088 | |
| Male, n(%) | 0.483 | 0.654-2.454 |  | |  |
| BMI, kg/m^2^ | 0.569 | 0.890-1.066 |  | |  |
| ACS, n(%): | 0.101 | 0.939-2.012 |  | |  |
| Smoking, n(%) | 0.379 | 0.429-1.380 |  | |  |
| Old stroke, n(%) | 0.475 | 0.144-2.460 |  | |  |
| Hypertension, n(%) | 0.226 | 0.786-2.765 |  | |  |
| Coronary artery disease, n (%) | 0.002 | 1.415-4.987 | 0.025 | 1.096-3.948 | |
| Congestive heart failure, n (%) | 0.631 | 0-11239.113 |  | |  |
| Duration of T2DM, years | 0.147 | 0.990-1.067 |  | |  |
| Gensini score | 0.436 | 0.988-1.005 |  | |  |
| Coronary stenting, n(%) | 0.743 | 0.510-2.566 |  | |  |
| β-Blocker, n(%) | 0.323 | 0.671-3.360 |  | |  |
| ACEi/ARB/ARNi, n(%) | 0.611 | 0.605-2.351 |  | |  |
| SGLT-2i, n(%) | 0.201 | 0.802-2.854 |  | | - |
| CCB, n(%) | 0.818 | 0.353-2.277 |  | |  |
| Diuretics, n(%) | 0.307 | 0.735-2.666 |  | |  |
| Insulin, n(%) | 0.555 | 0.644-2.268 |  | |  |
| Sulfonylurea, n(%) | 0.898 | 0.470-2.363 |  | |  |
| GLP-1 receptor agonist, n(%) | 0.698 | 0.474-3.049 |  | |  |
| DPP-IV Inhibitors, n(%) | 0.589 | 0.235-12.766 |  | |  |
| Metformin, n(%) | 0.238 | 0.392-1.261 |  | |  |
| HbA1c | 0.642 | 0.872-1.249 |  | |  |
| LVEF (%) | 0.175 | 0.952-1.009 |  | |  |
| LAD (mm) | 0.004 | 1.025-1.137 | 0.029 | 1.006-1.114 | |
| LVIDd (mm) | 0.137 | 0.987-1.099 |  | |  |
| IVSd (mm) | 0.190 | 0.915-1.565 |  | |  |
| LVPWd (mm) | 0.158 | 0.917-1.707 |  | |  |

**Table S2 Univariate and multivariate analyses of rehospitalization for ACS or HF**

|  | Univariate Analysis | | Multivariate Analysis | |
| --- | --- | --- | --- | --- |
|  | *P*-value | 95% CI | *P*-value | 95% CI |
| Age, years | 0.001 | 1.024-1.101 | 0.014 | 1.010-1.091 |
| Male, n(%) | 0.380 | 0.692-2.626 |  |  |
| BMI, kg/m^2^ | 0.784 | 0.901-1.082 |  |  |
| ACS, n(%): | 0.193 | 0.877-1.918 |  |  |
| Smoking, n(%) | 0.405 | 0.426-1.411 |  |  |
| Old stroke, n(%) | 0.516 | 0.151-2.583 |  |  |
| Hypertension*, n(%) | 0.194 | 0.803-2.938 | 0.231 | 0.769-2.964 |
| Coronary artery disease, n (%) | 0.005 | 1.323-4.850 | 0.069 | 0.954-3.606 |
| Congestive heart failure, n (%) | 0.641 | 0-16126.526 |  |  |
| Duration of T2DM, years | 0.173 | 0.988-1.067 |  |  |
| Gensini score | 0.520 | 0.989-1.006 |  |  |
| Coronary stenting, n(%) | 0.848 | 0.481-2.436 |  |  |
| β-Blocker, n (%) | 0.221 | 0.723-4.057 |  |  |
| ACEi/ARB/ARNi, n(%) | 0.743 | 0.566-2.221 |  |  |
| SGLT-2i, n(%) | 0.095 | 0.897-3.373 | 0.093 | 0.284-1.102 |
| CCB, n(%) | 0.898 | 0.369-2.396 |  |  |
| Diuretics, n(%) | 0.402 | 0.683-2.589 |  |  |
| Insulin, n(%) | 0.919 | 0.533-2.012 |  |  |
| Sulfonylurea, n(%) | 0.852 | 0.388-2.184 |  |  |
| GLP-1 receptor agonist, n(%) | 0.622 | 0.497-3.216 |  |  |
| DPP-IV Inhibitors, n(%) | 0.555 | 0.247-13.486 |  |  |
| Metformin, n(%) | 0.397 | 0.414-1.405 |  |  |
| HbA1c | 0.804 | 0.850-1.233 |  |  |
| LVEF (%) | 0.704 | 0.963-1.026 |  |  |
| LAD (mm) | 0.004 | 1.025-1.139 | 0.019 | 1.010-1.119 |
| LVIDd (mm) | 0.507 | 0.962-1.081 |  |  |
| IVSd (mm) | 0.175 | 0.919-1.590 |  |  |
| LVPWd (mm) | 0.113 | 0.941-1.770 |  |  |

**: Hypertension was incorporated as a confounding factor in the multivariable regression model to eliminate the influence of differences in hypertension prevalence between both groups.*

**Table S3 Multivariate analyses of each component of rehospitalization**

|  | Rehospitalization for ACS | | Rehospitalization for HF | |
| --- | --- | --- | --- | --- |
|  | *P*-value | 95% CI | *P*-value | 95% CI |
| Age, years | 0.133 | 0.990-1.076 | 0.007 | 1.036-1.253 |
| Male, n(%) |  |  |  |  |
| BMI, kg/m^2^ |  |  |  |  |
| ACS, n(%): |  |  |  |  |
| Smoking, n(%) |  |  |  |  |
| Old stroke, n(%) |  |  |  |  |
| Hypertension*, n(%) |  |  | 0.522 | 0.221-2.149 |
| Coronary artery disease, n (%) | 0.070 | 0.942-4.617 | 0.685 | 0.379-4.383 |
| Congestive heart failure, n (%) |  |  |  |  |
| Duration of T2DM, years |  |  |  |  |
| Gensini score |  |  |  |  |
| Coronary stenting, n(%) |  |  |  |  |
| β-Blocker, n(%) |  |  |  |  |
| ACEi/ARB/ARNi, n(%) |  |  |  |  |
| SGLT-2i, n(%) |  |  | 0.045 | 0.043-0.964 |
| CCB, n(%) |  |  |  |  |
| Diuretics, n(%) |  |  |  |  |
| Insulin, n(%) |  |  |  |  |
| Sulfonylurea, n(%) |  |  |  |  |
| GLP-1 receptor agonist, n(%) |  |  |  |  |
| DPP-IV Inhibitors, n(%) |  |  |  |  |
| Metformin, n(%) |  |  |  |  |
| HbA1c |  |  |  |  |
| LVEF (%) |  |  |  |  |
| LAD (mm) | 0.166 | 0.981-1.118 | 0.060 | 0.997-1.171 |
| LVIDd (mm) |  |  |  |  |
| IVSd (mm) |  |  |  |  |
| LVPWd (mm) |  |  |  |  |

**: Hypertension was incorporated as a confounding factor in the multivariable regression model to eliminate the influence of differences in hypertension prevalence between both groups.*

**Table S4 Univariate and multivariate analyses of cardiovascular mortality**

|  | Univariate Analysis | | Multivariate Analysis | |
| --- | --- | --- | --- | --- |
|  | *P*-value | 95% CI | *P*-value | 95% CI |
| Age, years | 0.442 | 0.940-1.151 |  |  |
| Male, n(%) | 0.354 | 0.389-14.008 |  |  |
| BMI, kg/m^2^ | 0.005 | 0.436-0.864 | 0.604 | 0-149.547 |
| ACS, n(%): | 0.116 | 0.790-8.408 |  |  |
| Smoking, n(%) | 0.363 | 0.072-2.617 |  |  |
| Old stroke, n(%) | 0.684 | 0-147016.674 |  |  |
| Hypertension, n(%) | 0.356 | 0.072-2.576 |  |  |
| Coronary artery disease, n (%) | 0.004 | 2.707-217.455 | 0.886 | 0-2.926E+13 |
| Congestive heart failure, n (%) | 0.866 | 0-860234.123 |  |  |
| Duration of T2DM, years | 0.079 | 0.990-1.200 | 0.972 | 0.022-52.694 |
| Gensini score | 0.168 | 0.928-1.013 |  |  |
| Coronary stenting, n(%) | 0.038 | 0.025-0.899 | 0.656 | 0-155086125.6 |
| β-Blocker, n(%) | 0.306 | 0.065-2.356 |  |  |
| ACEi/ARB/ARNi, n(%) | 0.667 | 0.181-14.491 |  |  |
| SGLT-2i, n(%) | 0.548 | 0.280-10.972 |  |  |
| CCB, n(%) | 0.601 | 0-6451.433 |  |  |
| Diuretics, n(%) | 0.352 | 0.390-14.038 |  |  |
| Insulin, n(%) | 0.116 | 0.702-25.176 |  |  |
| Sulfonylurea, n(%) | 0.117 | 0.699-25.218 |  |  |
| GLP-1 receptor agonist, n(%) | 0.637 | 0-20640.114 |  |  |
| DPP-IV Inhibitors, n(%) | 0.857 | 0-9.930E+12 |  |  |
| Metformin, n(%) | 0.111 | 0.019-1.511 |  |  |
| HbA1c | 0.010 | 1.163-3.092 | 0.734 | 0-2935313.706 |
| LVEF (%) | 0.001 | 0.808-0.945 | 0.799 | 0.046-10.798 |
| LAD (mm) | 0.327 | 0.924-1.269 |  |  |
| LVIDd (mm) | 0.010 | 1.040-1.326 | 0.831 | 0.003-1279.197 |
| IVSd (mm) | 0.259 | 0.264-1.430 |  |  |
| LVPWd (mm) | 0.046 | 0.057-0.973 | 0.915 | 0-1.968E+17 |

**Table S5 Univariate and multivariate analyses of angina symptom control rate**

|  | Univariate Analysis | | Multivariate Analysis | |
| --- | --- | --- | --- | --- |
|  | *P*-value | 95% CI | *P*-value | 95% CI |
| Age, years | 0.192 | 0.988-1.064 |  |  |
| sex, n(%) | 0.072 | 0.940-4.300 | 0.180 | 0.768-4.090 |
| BMI, kg/m^2^ | 0.269 | 0.957-1.170 |  |  |
| ACS, n(%): | 0.588 | 0.751-1.658 |  |  |
| Smoking, n(%) | 0.408 | 0.375-1.490 |  |  |
| Old stroke, n(%) | 0.321 | 0.044-2.776 |  |  |
| Hypertension, n(%) | 0.057 | 0.979-4.620 | 0.081 | 0.914-4.830 |
| Coronary artery disease, n (%) | 0.063 | 0.958-5.132 | 0.140 | 0.804-4.754 |
| Congestive heart failure, n (%) | 0.999 | / |  |  |
| Duration of T2DM, years | 0.091 | 0.993-1.092 | 0.473 | 0.967-1.074 |
| Gensini score | 0.312 | 0.985-1.005 |  |  |
| Coronary stenting, n(%) | 0.200 | 0.707-5.258 |  |  |
| β-Blocker, n(%) | 0.940 | 0.438-2.146 |  |  |
| ACEi/ARB/ARNi, n(%) | 0.784 | 0.424-1.910 |  |  |
| SGLT-2i, n(%) | 0.059 | 0.255-1.025 | 0.031 | 0.213-0.931 |
| CCB, n(%) | 0.779 | 0.309-2.413 |  |  |
| Diuretics, n(%) | 0.589 | 0.547-2.894 |  |  |
| Insulin, n(%) | 0.959 | 0.453-2.210 |  |  |
| Sulfonylurea, n(%) | 0.597 | 0.489-3.471 |  |  |
| GLP-1 receptor agonist, n(%) | 0.126 | 0.809-5.562 |  |  |
| DPP-IV Inhibitors, n(%) | 0.931 | 0.120-10.139 |  |  |
| Metformin, n(%) | 0.530 | 0.403-1.597 |  |  |
| HbA1c | 0.654 | 0.757-1.191 |  |  |
| LVEF (%) | 0.421 | 0.979-1.053 |  |  |
| LAD (mm) | 0.055 | 0.998-1.172 | 0.068 | 0.994-1.178 |
| LVIDd (mm) | 0.867 | 0.938-1.079 |  |  |
| IVSd (mm) | 0.761 | 0.756-1.466 |  |  |
| LVPWd (mm) | 0.609 | 0.752-1.626 |  |  |
